# Supplementary material for: FERN – a Java framework for stochastic simulation and evaluation of reaction networks
Source: BMC Bioinformatics. 2008 Aug 29;9:356. doi: 10.1186/1471-2105-9-356 (PMC2553347; doi:10.1186/1471-2105-9-356)
Supplement: Additional file 1 — FERN distribution, Version 1.3. This archive contains the FERN source code and binaries as well as documentation and example models in FernML and SBML. [file 1471-2105-9-356-S1.zip › fern/doc/javadoc/fern/tools/gnuplot/package-tree.html]

fern.tools.gnuplot Class Hierarchy


---


|  |  |  |
| --- | --- | --- |
| **Overview** \| **Package** \| Class \| Use \| **Tree** \| **Deprecated** \| **Index** \| **Help** | |  |
| **PREV**   NEXT | **FRAMES**    **NO FRAMES**     **All Classes** |  |


---


## Hierarchy For Package fern.tools.gnuplot

**Package Hierarchies:**: All Packages

---

## Class Hierarchy

- java.lang.**Object**
  - fern.tools.gnuplot.**Axes** (implements java.lang.Iterable<T>)
    - fern.tools.gnuplot.**ArrayMatrixAxes**
      - fern.tools.gnuplot.**TransposedArrayMatrixAxes**
      - fern.tools.gnuplot.**CollectionAxes**
    - java.awt.**Component** (implements java.awt.image.ImageObserver, java.awt.MenuContainer, java.io.Serializable)
      - java.awt.**Container**
        - java.awt.**Window** (implements javax.accessibility.Accessible)
          - java.awt.**Frame** (implements java.awt.MenuContainer)
            - javax.swing.**JFrame** (implements javax.accessibility.Accessible, javax.swing.RootPaneContainer, javax.swing.WindowConstants)
              - fern.tools.gnuplot.**GnuPlot**

---


|  |  |  |
| --- | --- | --- |
| **Overview** \| **Package** \| Class \| Use \| **Tree** \| **Deprecated** \| **Index** \| **Help** | |  |
| **PREV**   NEXT | **FRAMES**    **NO FRAMES**     **All Classes** |  |


---
